# *PKD1* Duplicated regions limit clinical Utility of Whole Exome Sequencing for Genetic Diagnosis of Autosomal Dominant Polycystic Kidney Disease

**DOI:** 10.1038/s41598-019-40761-w

**Published:** 2019-03-11

**Authors:** Hamad Ali, Fahd Al-Mulla, Naser Hussain, Medhat Naim, Akram M. Asbeutah, Ali AlSahow, Mohamed Abu-Farha, Jehad Abubaker, Ashraf Al Madhoun, Sajjad Ahmad, Peter C. Harris

**Affiliations:** 1 Department of Medical Laboratory Sciences, Faculty of Allied Health Sciences, Health Sciences Center, Kuwait University, Jabriya, Kuwait; 2 Department of Genetics and Bioinformatics, Dasman Diabetes Institute (DDI), Dasman, Kuwait; 3 Division of Nephrology, Mubarak Al-Kabeer Hospital, Ministry of Health, Jabriya, Kuwait; 4 Department of Radiological Sciences, Faculty of Allied Health Sciences, Health Sciences Center, Kuwait University, Jabriya, Kuwait; 5 Division of Nephrology, Al-Jahra Hospital, Ministry of Health, Al-Jahra, Kuwait; 6 Department of Biochemistry and Molecular Biology, Dasman Diabetes Institute (DDI), Dasman, Kuwait; 7 Department of Cornea and External Diseases, Moorfields Eye Hospital-NHS Foundation Trust, London, United Kingdom; 8 Institute of Ophthalmology, University Collage London (UCL), London, United Kingdom; 9 Division of Nephrology and Hypertension, Mayo Clinic, Rochester, USA

## Abstract

Autosomal dominant polycystic kidney disease (ADPKD) is an inherited monogenic renal disease characterised by the accumulation of clusters of fluid-filled cysts in the kidneys and is caused by mutations in *PKD1* or *PKD2* genes. ADPKD genetic diagnosis is complicated by *PKD1* pseudogenes located proximal to the original gene with a high degree of homology. The next generation sequencing (NGS) technology including whole exome sequencing (WES) and whole genome sequencing (WGS), is becoming more affordable and its use in the detection of ADPKD mutations for diagnostic and research purposes more widespread. However, how well does NGS technology compare with the Gold standard (Sanger sequencing) in the detection of ADPKD mutations? Is a question that remains to be answered. We have evaluated the efficacy of WES, WGS and targeted enrichment methodologies in detecting ADPKD mutations in the *PKD1* and *PKD2* genes in patients who were clinically evaluated by ultrasonography and renal function tests. Our results showed that WES detected *PKD1* mutations in ADPKD patients with 50% sensitivity, as the reading depth and sequencing quality were low in the duplicated regions of PKD1 (exons 1–32) compared with those of WGS and target enrichment arrays. Our investigation highlights major limitations of WES in ADPKD genetic diagnosis. Enhancing reading depth, quality and sensitivity of WES in the *PKD1* duplicated regions (exons 1–32) is crucial for its potential diagnostic or research applications.

## Introduction

Autosomal dominant polycystic kidney disease (ADPKD) is an inherited renal disease characterised by the accumulation of clusters of fluid-filled cysts in the kidneys, with reported incidence ranging between 1:400 and 1:1000 worldwide^1^. It is a progressive, monogenic disease that impairs kidney function and eventually causes end-stage renal disease (ESRD). In addition, ADPKD patients may develop extrarenal manifestations, including hepatic and pancreatic cysts, cerebral and aortic aneurysms, cardiac valvular abnormalities and hypertension^2^.

ADPKD is primarily caused by mutations in 2 genes: *PKD1* (MIM 601313) and *PKD2* (MIM 173910). Mutations in *PKD1*, which is located on chromosome 16 (16p13.3), account for approximately 85% of genetically resolved ADPKD cases, whereas mutations in *PKD2*, located on chromosome 4 (4q21-11), account for the remaining 15%^3^. *PKD1* encodes polycystin-1, which is an integral membrane protein that complexes with polycystin-2, a calcium-permeable cation channel involved in intracellular Ca^2+^ homoeostasis. The polycystin proteins form a functional complex in kidney tubular primary cilia, which is believed to be involved in adhesion, proliferation and differentiation of tubular epithelial cells^4^.

ADPKD shows some degree of complexity suggested by the observed heterogeneity at the genic and allelic levels^5,6^. At the genic level, mutations in *PKD1* are associated with a more severe disease phenotype and earlier age of ESRD onset compared with mutations in *PKD2* (54.3 years for PKD1 and 74 years for PKD2)^7,8^. At the allelic level, truncating mutations are associated with earlier mean age of ESRD onset than non-truncating mutations (55.6 and 67.9 years, respectively)^9^.

Genetic analysis and mutation screening of ADPKD cases is more technically challenging than that of other monogenic diseases. One reason is that both *PKD1* and *PKD2* are highly variable genes, and molecular analysis reveals no clear hot spots where mutations are likely to occur along the entire length of either gene^3,10^. Mutations in these genes are often unique to a single family, with recurrent mutations accounting for only 30% of the total detected mutations^11–15^. The autosomal dominant polycystic kidney disease mutation database (PKDB) established by the Mayo Clinic lists 1243 definite pathogenic *PKD1* variants out of the 2055 listed variants and 374 definite pathogenic *PKD2* variants out of the 463 listed variants (http://pkdb.mayo.edu). In addition, the existence of pseudogenes for *PKD1* results in another level of complexity for ADPKD genetic testing. *PKD1* lies in a segmentally duplicated region, such that the first 32 exons are replicated 6 times in pseudogenes located in a region 13–16 Mb proximal to the original *PKD1* (16p.13.1), and they share 97.6–97.8% sequence homology with the original *PKD1* gene^16–18^. As these pseudogene regions are less amenable to selection pressure, they tend to have high mutation rates compared with those of the parent gene^19^. These duplicated regions represent a diagnostic challenge for ADPKD, as conventional sequencing is not effective for specifically targeting the genuine *PKD1* regions^20^. To overcome this issue, novel sequencing strategies have been developed by utilising the rare sequence variations between the pseudogenes and original *PKD1* to specifically target exons 1–32 of the genuine *PKD1* gene. These techniques utilised long-range PCR (LR-PCR) to specifically generate amplicons from genuine regions and exclude the pseudo-regions, providing a more reliable molecular diagnostic tool for ADPKD^11,21^. ADPKD genetic diagnosis may complement the current diagnosis protocol, which relies primarily on ultrasonography^22^, particularly for younger, at-risk individuals as well as those with late onset disease for whom imaging-based diagnosis may be equivocal.

Since the completion of the human genome project, the demand for faster and more economical sequencing methods has led to the development of next generation sequencing (NGS) technologies as alternative/complementary methods to traditional testing. The massively parallel sequencing platforms of NGS facilitate faster sequencing with higher DNA throughput at lower cost than traditional Sanger sequencing^23,24^. This encouraged wider utilisation of technologies such as whole genome sequencing (WGS) and whole exome sequencing (WES) in medical diagnosis and research. While the human exome includes <2% of the whole genome, approximately 85% of disease-causing mutations and functional variants are located within these coding regions^25,26^. This makes WES more cost-effective than WGS in the identification of rare causes of genetic diseases as well as predisposing variants in known diseases and disorders^25^. ADPKD is one of the diseases that could benefit from NGS in the clinical settings. Moreover, this monogenic condition is considerably associated with intrafamilial phenotypic variability of the disease progression rate and the extrarenal manifestations, despite sharing the same mutation^5^. Such observations within ADPKD families suggest the involvement of heritable modifier genes^5,6^. WES and WGS may help in uncovering the modifier genes responsible for the significant phenotypic variability observed, which may aid in better understanding of the disease pathology. However, the reliability and efficiency of NGS-based strategies as diagnostic tools for ADPKD are yet to be examined and evaluated, specifically for the complex *PKD1* gene in terms of its large size, the absence of mutation hot spots and the existence of the *PKD1* duplicated regions—the pseudogenes^16,18^. Numerous studies have demonstrated that the high level of sequence similarity between the pseudogene sequences and their parent genes can obscure the detection of the pathogenic mutations, as the unintended detection of pseudo-mutations is possible^21,27^. Such false-positive results could affect the specificity and reliability of the analysis; therefore, it is essential to evaluate the clinical utility of NGS strategies for the diagnosis of ADPKD.

In the current study, we evaluated the efficiency of WES, WGS and targeted enrichment for sequencing *PKD1* and *PKD2* to detect ADPKD mutations in patients who have been clinically evaluated by ultrasonography, renal function analysis and long-range PCR (LR-PCR). Our results highlight major limitations of WES in the detection of *PKD1* mutations in ADPKD patients.

## Results

### Clinical evaluation revealed typical ADPKD symptoms

In the current study, total 51 individuals from 6 typical ADPKD families were enrolled. Twenty-six individuals had already been diagnosed with ADPKD according to the unified criteria for ultrasonographic diagnosis of ADPKD (Supplementary Fig. 1)^16^. The other 25 individuals were at risk, and their disease status was unknown when they consented to participate in the study. Following ultrasonographic analysis and RFTs, 7 individuals were diagnosed with ADPKD, whereas the remaining 18 individuals were healthy. All results were confirmed by genetic testing using Sanger sequencing of *PKD1* and *PKD2*.

### Detection of *PKD1* mutations using LR-PCR and Sanger sequencing

**Figure 1:**
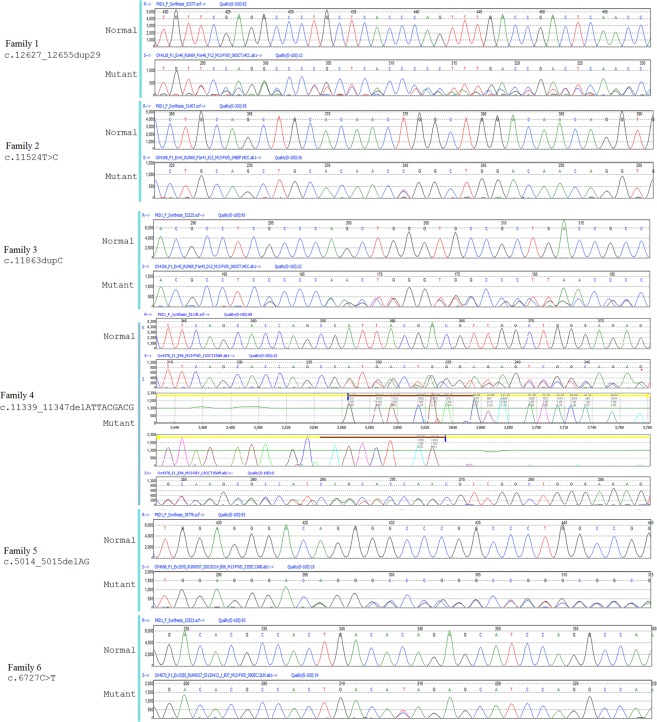
PKD1 mutations detected using targeted sequencing. A total of 6 mutations were found, 4 of which are novel.

Genetic analysis of the enrolled subjects revealed 6 *PKD1* mutations that segregated with the disease in the 6 ADPKD families included in the current study (Fig. 1 and Table 1). Four of the 6 mutations found are novel *PKD1* mutations. In family 1, we detected a novel duplication in exon 46; a stretch of 29 bases from position 12627 to 12655 were duplicated, causing a frameshift after position 12655 (c.12627_12655dup; Phe4219Cysfs). In family 2, we detected a novel missense mutation in exon 41 caused by substitution of thymidine to cytosine at position 11524 that results in tryptophan substitution with arginine at position 3842 of the protein (c11524T > C; p.Trp3842Arg). The mutation showed a score of 1 using Polyphen-2 and 0 using SIFT, which suggests pathological significance. In family 3, we detected a novel *PKD1* mutation in exon 41 caused by duplication of the cytosine at position 11863, resulting in a frameshift at position 3955 that leads to a premature stop codon because of the substituted amino acid at position 6 (c.11863dupC, p.Gln3955Profs*6). As this mutation is predicted to cause a frameshift, we define it as pathogenic. In family 4, a novel deletion of 9 bases was detected in exon 40 of *PKD1* (c.11339_11347del9), resulting in the deletion of 3 amino acids from the protein (p.Asp3780_D3782del). In family 5, we detected a two-base deletion mutation in exon 15 (c.5014_5015delAG). This deletion caused a frameshift from position 1672, leading to a premature stop codon at position 98 after the substitution (p.Arg1672fs98*). This particular mutation is the most frequent mutation detected in ADPKD families^9,15^. In family 6, a known nonsense mutation was detected in exon 15. This truncating mutation resulted from a single base change (c.6727 C > T), causing a premature stop codon p.Gln2243*^10,28^.

**Table 1:** ADPKD mutations detected by LR-PCR and Sanger’s sequencing.

Family	Exon	Variant DNA	Variant Protein	Mutation Type	Reference
1	46	c.12627_12655dup29	p.Phe4219fs	Duplication/Frameshift	Novel
2	41	c.11524 T > C	p.Trp3842Arg	Missense	Novel
3	43	c.11863dupC	p.Gln3955fs*6	Duplication/Frameshift	Novel
4	40	c.11339_11347delATTACGACG	p.Asp3780_Asp3782del	Deletion/Framshift	Novel
5	15†	**c.5014_5015delAG**	p.Arg1672Glyfs*98	**Deletion/Frameshift**	**[15]**
6	15	c.6727 C > T	p.Gln2243*	Nonsense	[28]

^†^Mutation could not be detected by WES.

### Identification of *PKD1* and *PKD2* variants using WES

WES of the samples from the 26 ADPKD patients (known to have ADPKD before study enrolment) identified 27 variants in *PKD1* (including 6 novel variants) and 5 variants in *PKD2*. Of the 27 *PKD1* variants, 12 were synonymous changes, 12 were non-synonymous changes and the remaining 3 were insertions/deletions. For *PKD2* variants, only 1 synonymous and 4 non-synonymous changes were identified (Table 2). Five of the 6 ADPKD mutations identified using Sanger sequencing were identified by WES, whereas the remaining mutations (c.5014_5015delAG, found in family 5) could not be detected in the 8 patients included in WES analysis from this family (Tables 1 and 2). In addition, the mutation found in family 6 (16:2158441-SNV, c.6727 C > T) was detected only in one patient out of 6 included in the WES analysis from this family. While 4 of the 5 ADPKD mutations detected using WES were frameshift insertions and deletions, the remaining reported mutation was a missense mutation (16:2141795-SNV, c.11524 T > C; p.Trp3842Arg), and its pathological impact was evaluated and confirmed using the pathological prediction and conservation scores (Supplementary Tables 1,2).

**Table 2:** *PKD1* and *PKD2* variants detected by WES. ADPKD mutations highlighted in bold.

WES Variant	Exon	Classification	cDNA Change	Protein Change	rs ID
*PKD1* (NM_001009944.2)					
**16:2140009-Ins**	**46**	**Frameshift Ins**	**c.12627_12655dup29**	**p.Glu4219fs**	**Novel**
16:2140010-MIX	46	Synonymous	c.12630 T > C	p.=	rs7203729
16:2140294-SNV	45	Nonsyn SNV	c.12436 G > A	p.Val4146Ile	rs148478410
16:2140321-SNV	45	Synonymous	c.12409 C > T	p.=	rs79899502
16:2140454-SNV	45	Synonymous	c.12276 A > G	p.=	rs3087632
16:2140554-SNV	45	Nonsyn SNV	c.12176 C > T	p.Ala4059Val	rs3209986
16:2140680-SNV	44	Nonsyn SNV	c.12133 A > G	p.Ile4045Val	rs10960
**16:2141025-Ins**	**43**	**Frameshift Ins**	**c.11863_11864dupC**	**p.Gln3955fs**	**Novel**
16:2141028-SNV	43	Nonsyn SNV	c.11860 G > C	p.Ala3954Pro	Novel
**16:2141795-SNV**	**41**	**Nonsyn SNV**	**c.11524 T > C**	**p.Trp3842Arg**	**Novel**
**16:2142112-Del**	**40**	**Del**	**c.11339_11347delATTACGACG**	**p.Asp3780_Asp3782del**	**Novel**
16:2142113-SNV	40	Synonymous	c.11346 C > T	p.=	rs145955373
16:2144176-SNV	35	Nonsyn SNV	c.10535 C > T	p.Ala3512Val	rs34197769
16:2144182-SNV	35	Nonsyn SNV	c.10529 C > T	p.Thr3510Met	rs45478794
16:2147399-SNV	33	Synonymous	c.10326 G > A	p.=	rs141138826
16:2152387-SNV	25	Nonsyn SNV	c.9196 T > C	p.Phe3066Leu	rs77028972
16:2152388-SNV	25	Synonymous	c.9195 G > C	p.=	rs78003543; rs9935834
16:2156447-SNV	18	Synonymous	c.7441 C > T	p.=	rs2003782
**16:2158441-SNV†**	**15**	**Stopgain**	**c.6727 C > T**	p.Gln2243*	^28^
16:2158570-SNV	15	Nonsyn SNV	c.6598 C > T	p.Arg2200Cys	rs140869992
16:2159405-SNV	15	Synonymous	c.5763 G > A	p.=	rs2575313
16:2159996-SNV	15	Synonymous	c.5172 C > T	p.=	rs9935526
16:2160503-SNV	15	Synonymous	c.4665 A > C	p.=	rs71385734
16:2162887-SNV	13	Synonymous	c.3063 T > C	p.=	rs2369068
16:2164294-SNV	11	Synonymous	c.2730 C > T	p.=	rs35965348
16:2164808-SNV	11	Nonsyn SNV	c.2216 G > A	p.Arg739Gln	rs40433
16:2165470-SNV	10	Nonsyn SNV	c.2006G > T	p.Cys669Phe	Novel
*PKD2* (NM_000297.3)					
4:88928968-SNV	1	Nonsyn SNV	c.83 G > C	p.Arg28Pro	rs1805044
4:88929305-SNV	1	Synonymous	c.420 G > A	p.=	rs2728118
4:88929453-SNV	1	Nonsyn SNV	c.568 G > A	p.Ala190Thr	rs117078377
4:88967919-SNV	6	Nonsyn SNV	c.1445 T > G	p.Phe482Cys	rs75762896
4:88989089-SNV	13	Nonsyn SNV	c.2398 A > C	p.Met800Leu	rs2234917

^†^WES detected the mutation in 1 patient from a total of 9 ADPKD patients from the same family. Sanger’s sequencing confirmed that all the 9 family patients are carrying the mutation.

Highlighted variants in bold are the ADPKD mutations detected by WES.

Overall, the sensitivity of WES for detecting *PKD1* mutations throughout the length of the gene, which reflects the ability of the test to correctly identify the true disease-causing mutations, was 50%; however, for mutations in exons 1–32, it was only 7.14% and for exons 33–46, it was 100%. As there were no false-positive results recorded, the specificity of WES for detection of *PKD1* mutations was 100% for the entire length of the gene. As all the ADPKD mutations in enrolled patients were found in *PKD1*, sensitivity and specificity for *PKD2* could not be calculated (Table 3).

**Table 3:** Sensitivity and Specificity of WES for detection of PKD1 mutations.

	Sensitivity	Specificity
*PKD1* exons 1–32	7.14%	100%
*PKD1* exons 33–46	100%	100%
Entire *PKD1*	50%	100%

### WES of exons 1–32 of *PKD1* showed low reading depth, genotype quality and quality

**Figure 2:**
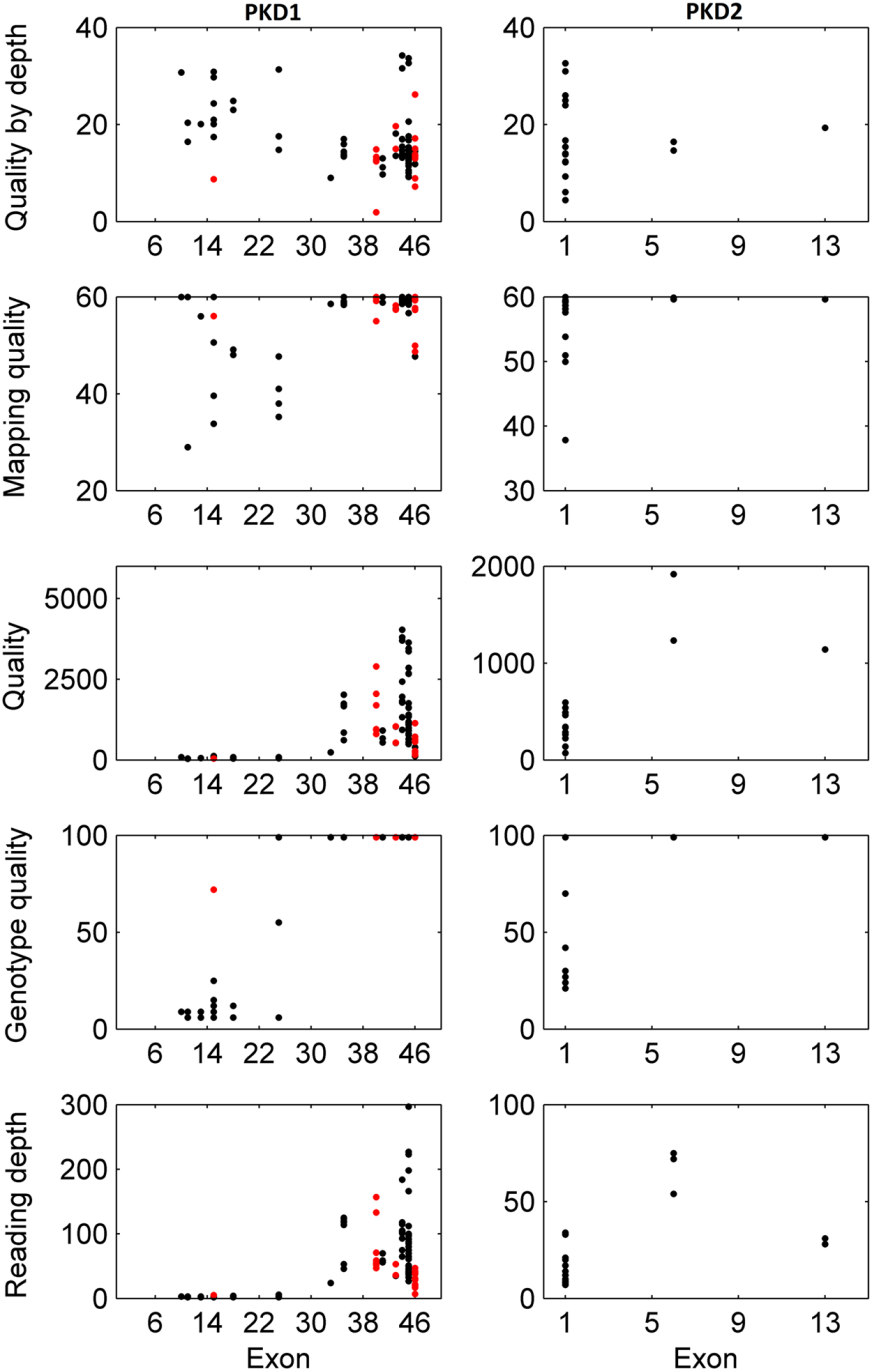
Quality assessment of called variants in PKD1 and PKD2 using WES. Exons 1–32 of PKD1 had low reading depth, genotype quality and quality. ADPKD mutations shown in red.

**Figure 3:**
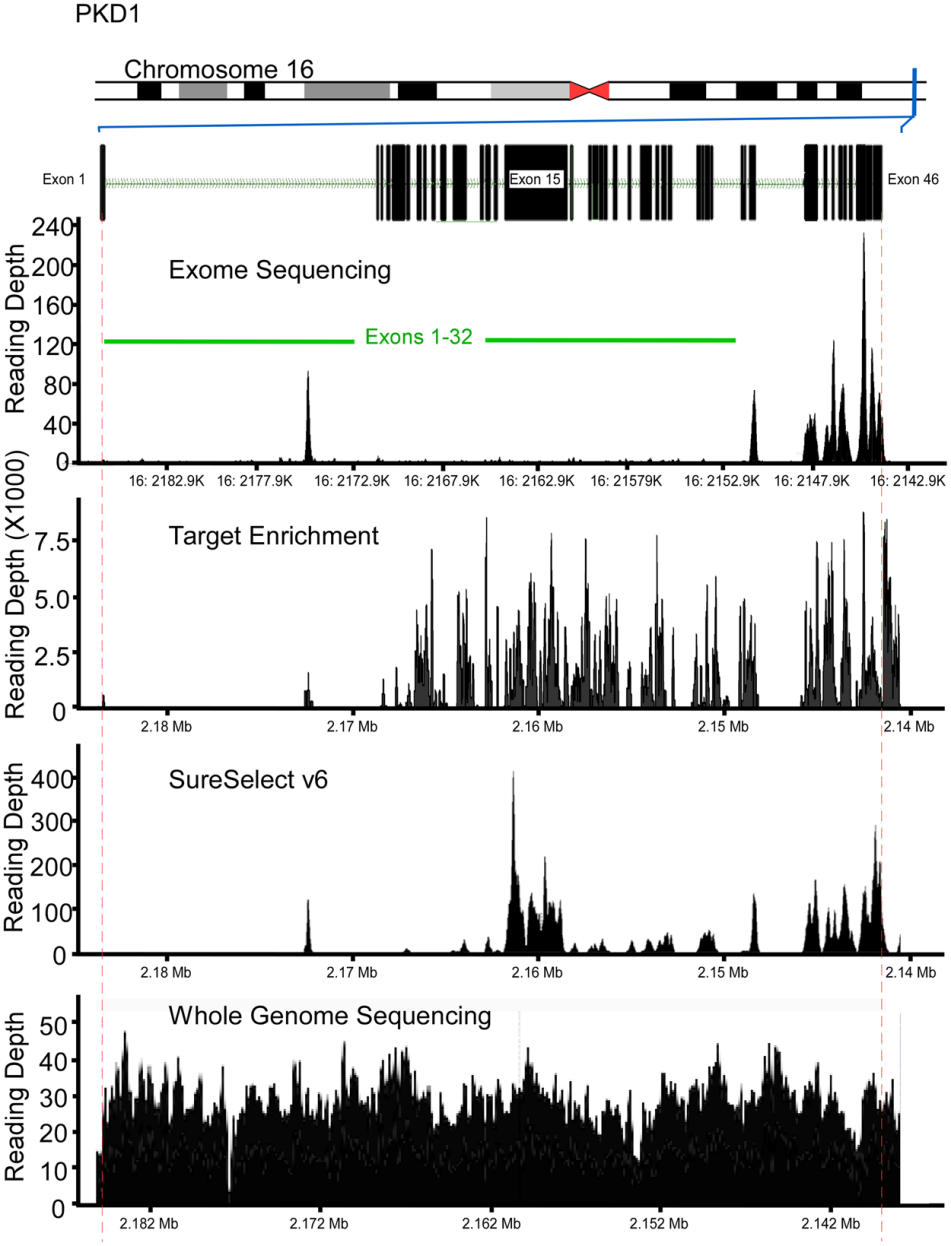
WES and Target enrichment coverage map of *PKD1*. WES of exons 1 to 32 of PKD1 showed low coverage while PKD2 coverage showed proper depth of all exons. Target Enrichment showed proper coverage for coding regions of PKD1 and PKD2. SureSelect v6 improved the coverage of PKD1 in comparison to WES TruSeq v3 but exons 1 to 14 remained poorly covered. WGS showed proper covering of the entire PKD1.

**Figure 4:**
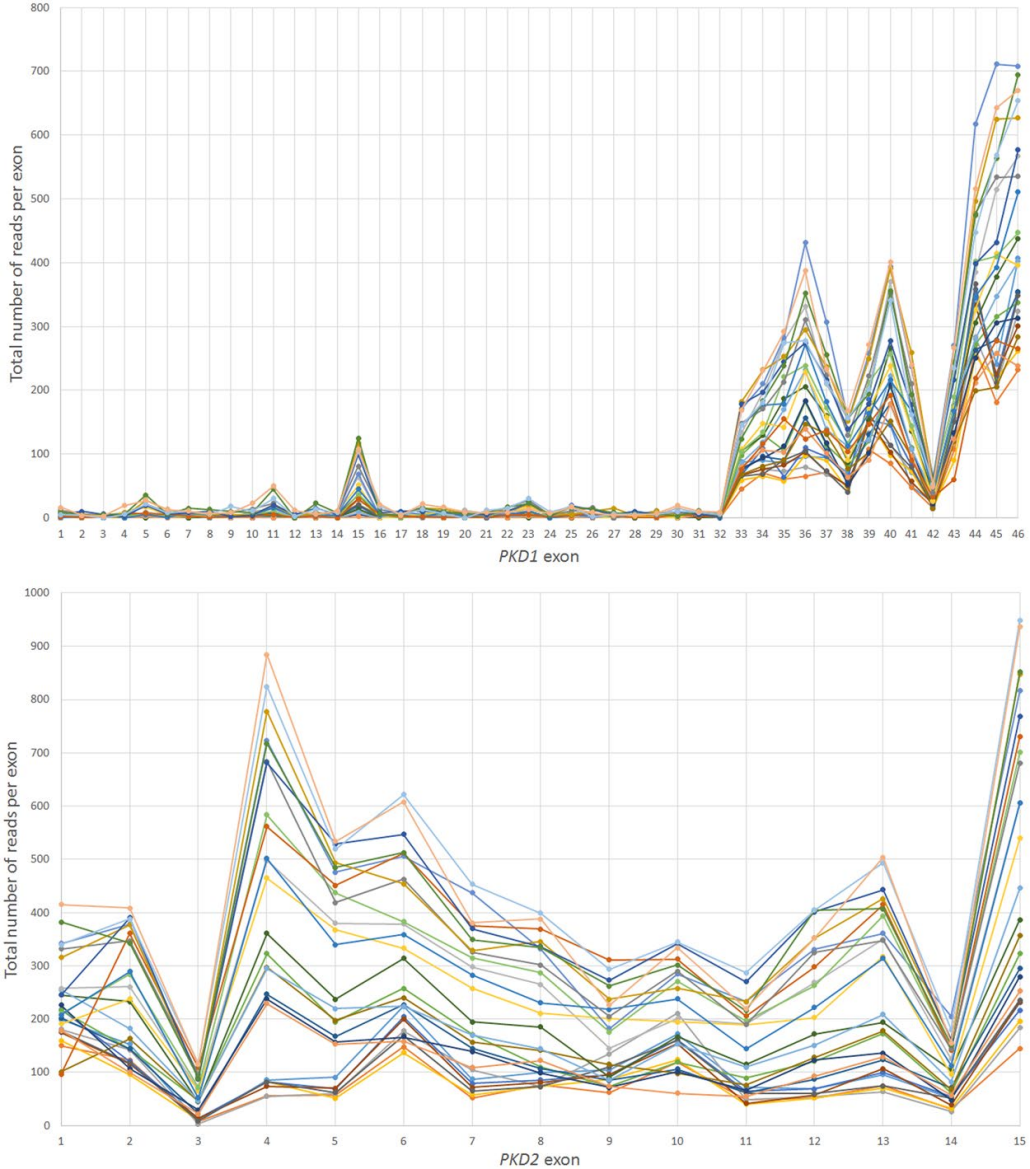
Total number of reads per exon for PKD1 and PKD2 using WES. Upper image shows that PKD1 exons 1 to 32 have relatively lower number of total reads in comparison with PKD1 exons 33–46 while PKD2 exons “lower image” shows better distribution of readings per exon in comparison with PKD1.

All variants detected in exons 1–32 of *PKD1* showed low mean reading depth (RD) ranging from 2 to 5 (Figs 2 and 3). Despite a low RD of 2, the mutation found in family 1 (16:2158441-SNV) was called and detected, but only in one of the 6 patients from this family (Tables 2 and 4). We also calculated the total number of reads per *PKD1* exon in each sample, and all samples showed a similar trend (Fig. 4). Exons 1–32 had a noticeably lower total number of reads in comparison to exons 33–46. Mean Genotype Quality (GQ) and mean quality were also low for variants called in exons 1–32 in comparison to variants called in exons 33–46. However, the mean Mapping Quality (MQ) was roughly the same for all variants called across the whole *PKD1* gene. When the quality was normalised by depth, called variants in exons 1–32 showed a relatively similar range of scores to those variants called in exons 33–46 (Fig. 2).

**Table 4:** Quality assessment of variants detected by WES.

WES Variant	Exon	Mean Reading Depth ( ± SD)	Mean Genotype Quality (GQ) ( ± SD)	Mean Mapping Quality (MQ) ( ± SD)	Mean Quality by Depth (QD) ( ± SD)		Mean Quality ( ± SD)
*PKD1* (NM_001009944.2)							
**16:2140009-Ins**	**46**	**14.0 (**±**7)**	**99 (**±**0)**	**48.2 (**±**0.73)**	**13.19 (**±**1.86)**		**255.23 (**±**193.04)**
16:2140010-MIX	46	29.33 (±11.68)	99 (±0)	56.08 (±4.71)	14.12 (±5.09)		524.63 (±312.54)
16:2140294-SNV	45	114.71 (±46.76)	99 (±0)	59.01 (±1.57)	11.34 (±2.09)		1924.45 (±1142.22)
16:2140321-SNV	45	216.40 (±44.17)	99 (±0)	59.05 (±0.69)	12.74 (±1.86)		2824.37 (±730.75)
16:2140454-SNV	45	53.60 (±19.97)	99 (±0)	59.41 (±0.49)	19.09 (±7.81)		1155.87 (±346.94)
16:2140554-SNV	45	55.80 (±11.33)	99 (±0)	59.37 (±0.61)	12.63 (±1.82)		708.17 (±135.65)
16:2140680-SNV	44	105 (±29.11)	99 (±0)	59.45 (±0.49)	18.99 (±8.04)		2420.89 (±1146.87)
**16:2141025-Ins**	**43**	**44.0 (**±**9)**	**99 (**±**0)**	**57.88 (**±**0.51)**	**15.86 (**±**3.22)**		**781.73 (**±**356.38)**
16:2141028-SNV	43	44.5 (±8.5)	99 (±0)	57.81 (±0.61)	17.32 (±0.3.32)		790.77 (±353.31)
**16:2141795-SNV**	**41**	**61.67 (**±**6.01)**	**99 (**±**0)**	**59.42 (**±**0.82)**	**11.32 (**±**1.65)**		**709.77 (**±**186.27)**
**16:2142112-Del**	**40**	**169 (NA)**	**99 (NA)**	**60 (NA)**	**17.12 (NA)**		**2894.73 (NA)**
16:2142113-SNV	40	67.14 (±27.77	99 (±0)	58.47 (±2.33)	13.17 (±1.02)		1290.31 (±550.12)
16:2144176-SNV	35	108 (±27.61)	99 (±0)	59.03 (±0.71)	14.99 (±1.44)		1612.17 (±446.16)
16:2144182-SNV	35	80 (±34)	99 (±0)	58.66 (±0.25)	13.88 (±0.67)		1141.27 (±741.75)
16:2147399-SNV	33	24 (NA)	99 (NA)	58.55 (NA)	9.78 (NA)		234.77 (NA)
16:2152387-SNV	25	3.25 (±1.64)	41.5 (±44.75)	40.45 (±6.45)	21.25 (±8.87)		68.09 (±18.58)
16:2152388-SNV	25	3.25 (±1.64)	41.5 (±44.75)	40.45 (±6.45)	21.25 (±8,87)		68.09 (±18.58)
16:2156447-SNV	18	3.0 (±1)	9 (±4.3)	48.14 (±0.79)	23.94 (±1.31)		70.88 (±29.90)
**16:2158441-SNV†**	**15**	**2.0 (NA)**	**6 (NA)**	**60 (NA)**	**30.87 (NA)**		**61.74 (NA)**
16:2158570-SNV	15	5.0 (NA)	72 (NA)	56.04 (NA)	8.75 (NA)		43.77 (NA)
16:2159405-SNV	15	3.0 (NA)	9 (NA)	60 (NA)	17.43 (NA)		52.28 (NA)
16:2159996-SNV	15	4.67 (±0.47)	17.3 (±6.8)	41.33 (±8.53)	25.03 (±4.42)		115.23 (±9.05)
16:2160503-SNV	15	3.0 (NA)	9 (NA)	60 (NA)	20.09 (NA)		60.28 (NA)
16:2162887-SNV	13	3.0 (NA)	9 (NA)	56 (NA)	20.09 (NA)		60.28 (NA)
16:2164294-SNV	11	3.0 (NA)	9 (NA)	60 (NA)	16.43 (NA)		49.28 (NA)
16:2164808-SNV	11	2.0 (NA)	6 (NA)	29 (NA)	20.35 (NA)		40.74 (NA)
16:2165470-SNV	10	3.0 (NA)	9 (NA)	60 (NA)	30.09 (NA)		92.28 (NA)
*PKD2* (NM_000297.3)							
4:88928968-SNV	1	12.6 (±5.35)	67.9 (±35.45)	53.31 (±7.45)	19.86 (±8.51)	322.69 (±125.66)	
4:88929305-SNV	1	27 (±11.26)	99 (NA)	59.32 (±0.16)	13.91 (±2.14)	567.04 (±38.47)	
4:88929453-SNV	1	11 (±1.41)	64.5 (±48.79)	59.64 (±0.51)	12.16 (±12.01)	179.42 (±150.83)	
4:88967919-SNV	6	67 (±11.35)	99 (NA)	59.755 (±0.17)	15.54 (±1.27)	1576.08 (±483.92)	
4:88989089-SNV	13	31 (NA)	99 (NA)	59.63 (NA)	36.77 (NA)	1140.41 (NA)	

Standard deviation (SD) is noted as non-applicable (NA) when there is only one value indicating that the variant was detected in one sample.

**Figure 5:**
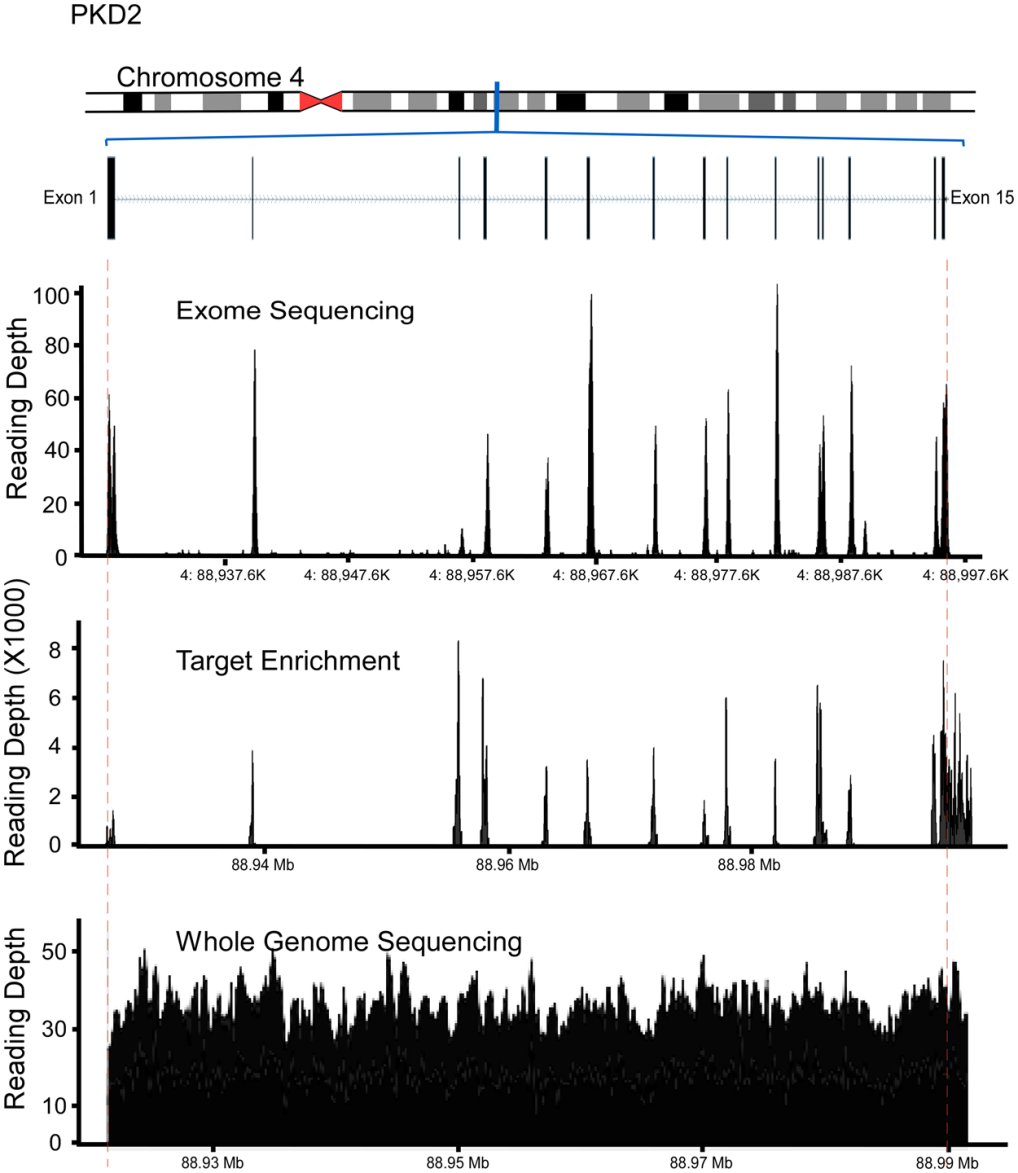
WES, Target enrichment coverage map and WGS of *PKD2. PKD2* was covered properly using all methods.

For *PKD2*, all exons were properly covered, and all called variants showed a mean RD > 10, mean GQ was >60, mean MQ was close to 60 and mean quality ranged between 179.42 and 1576.08. Exon 3 showed the lowest total number of reads in all samples (Figs 2, 4 and 5).

### WGS detected *PKD1* mutations located in the duplicated region

DNA samples of 4 ADPKD patients were selected for WGS analysis: 2 patients with *PKD1* mutations in the duplicated region of *PKD1* and 2 patients with mutations outside the duplicated region of *PKD1* (mutations were previously identified using LR-PCR). The coverage analysis of WGS for *PKD1* and *PKD2* showed good and uniform coverage, including exons 1–32 of *PKD1* (Figs 3 and 4). Three out of 4 mutations were successfully detected using WGS, including the 2 mutations located in the *PKD1* duplicated region (exon 15), c.5014_5015delAG and c.6727 C > T, whereas c.12627_12655dup29 (located in exon 46) was missed (Table 5).

**Table 5:** PKD1 mutations detected by WGS*.

cDNA Change	Protein	Mean Depth	Quality
c.11863dupC	p.Q3955fs	25	40.3
c.5014_5015delAG	p.Arg1672Glyfs*98	34	34.9
c.6727 C > T	p.Q2243X	37	40.1

*The genomes of 4 ADPKD with known mutations where analyzed using WGS. 3 out 4 mutations were detected successfully while the c.12627_12655dup29 was missed.

### Targeted enrichment system detected mutations in PKD1 duplicated regions

Samples with *PKD1* mutations from families 5 (c.5014_5015delAG) and 6 (c.6727 C > T) both located in exon 15, which were found using LR-PCR and Sanger sequencing but could not be detected with WES, were reanalysed using targeted enrichment arrays. These specific mutations were identified in all the samples analysed using targeted enrichment of *PKD1*. Coverage maps of targeted enrichment of *PKD1* and *PKD2* showed good coverage and depth in the coding regions of both genes (Figs 3 and 5). The newer version of SureSelect capture arrays shows significant improvement in the representation of exons 15–32 of the *PKD1* gene (Fig. 3). However, capture of exons 1–14 remains poor.

## Discussion

Mutations in either *PKD1* or *PKD2* may cause ADPKD. While genetic analysis of *PKD2* is relatively easy, genetic analysis of *PKD1* is more complex because of its highly polymorphic nature, large size and pseudogene regions. LR-PCR and Sanger sequencing are the current gold standard methods for genetic analysis of *PKD1*. However, these methods are labour intensive and require a substantial amount of time to analyse the large number of amplicons. In the current study, we evaluated the efficiency of WES, WGS and target enrichment as genetic diagnostic tools for ADPKD and as potential replacement methods for the LR-PCR and Sanger sequencing techniques.

ADPKD is diagnosed using ultrasonographic age-related cyst number criteria^29^. It has also been shown that computed tomography and magnetic resonance imaging may be effectively utilised for the same purpose^30^. These image-based diagnostic approaches are highly reliable in older patients (aged >30 years) but not in younger adult patients, which limits their utility in kidney donations to exclude disease status in young kidney donors^11^. Genetic analysis of ADPKD genes provides an alternative reliable diagnosis tool for younger patients. In the current study, these methods demonstrated high sensitivity and specificity, as they detected all the pathogenic mutations that segregated with the disease in all patients.

Recent advances in genetic sequencing, including NGS platforms such as WES, are considered revolutionising tools for genetic diagnostic research. The ability of WES to generate accurate, efficient, fast and cost-effective genetic data is important when considering implementation in medical practice to improve diagnosis and disease treatments in general. For ADPKD, WES is considered an effective tool to explore and identify potential disease modifier genes, particularly where our current knowledge of these modifiers is poor^31–33^. In this study, we assessed the effectiveness of utilising WES as a diagnostic tool for ADPKD and whether it can replace the current gold standard methods for ADPKD genetic diagnosis: LR-PCR and Sanger sequencing. To achieve this objective, we avoided filtering the called variants and analysed the quality scores for all variants called in *PKD1* and *PKD2*. WES showed major limitations when used to detect mutations in *PKD1* exons 1–32, as test sensitivity for this particular region was only 7.14%, whereas it demonstrated high sensitivity for the remaining coding regions of *PKD1*. This was also reflected by the low RDs observed, particularly over *PKD1* exons 1–32 (Figs 2 and 3). The low RD concurred with the significantly lower number of total reads per exon in this region compared to the *PKD1* exons 33–46 and *PKD2* (Fig. 5). Moreover, the mean GQ for the called variants in *PKD1* exons 1–32 was correlated with the read depth pattern, as they scored low in comparison with the variants called elsewhere in *PKD1* and *PKD2*. GQ indicates the likelihood of the called genotype being correct; the higher the value, the more accurate the genotype calls. Normally, a GQ > 20 is considered acceptable^33^; however, in our WES results, the mean GQ for called variants in *PKD1* exons 1–32 ranged from 6 to 72 (Table 4). Despite the low GQ score of 6 and a RD of only 2 for the called mutation found in family 1 (16:2158441-SNV, c.6727 C > T), the WES results were confirmed by LR-PCR and Sanger sequencing, indicating its validity. However, the mean MQ scores across *PKD1* and *PKD2* were high in general, indicating that all the reads were informative and correctly mapped. Although there is no clear cut off MQ value^34^, a score > 20 is usually acceptable according to the GATK tool^35^ (Table 4). These poor results may be attributed to the *PKD1* pseudogenes, as designing capture oligonucleotide probes (65 nt) that specifically target *PKD1* genuine regions and avoid its pseudo-regions poses a considerable challenge. As a result, pseudogenes are captured in parallel with the genuine *PKD1* regions, complicating data analysis, compromising result reliability and often yielding false-positive results, as mutations in pseudogenes are detected, resulting in reduced test specificity^21,27,36^. However, it is possible that readings mapped to multiple genome sites tend to be avoided, as demonstrated by our results. In general, the low coverage and RD of the *PKD1* exons 1–32 could represent a major diagnostic limitation, as some mutations may be missed and consequently compromise the sensitivity and efficacy of the test. In both WES and WGS, mutation detection sensitivity can be improved by increasing the depth of sequencing and by the utilization of newer capture arrays. The newer version of SureSelect V6 capture arrays shows significant improvement in the representation of the exons 15–32 of the PKD1 gene (Fig. 3). For example, SureSelect V6 arrays covers all exons with a target size of 60 MB and 758,086 probes, while V4 of SureSelect capture arrays have a target size of 51 MB and covers 334,378 exons.

WGS showed a more uniform representation of entire *PKD1* and *PKD2*, including exons 1–32 of *PKD1*. It has been suggested that longer read lengths and avoidance of capture bias enhanced the ability of WGS to detect pathological ADPKD mutations, including those in the duplicated region of *PKD1*^37,38^. However, detecting small to medium size indels (10–1000 bases) remains a challenge (Table 5). We are currently utilizing three algorithms in parallel; Pindel, UnifiedGenotyper and HaplotypeCaller to call indels^39^.

Target enrichment of *PKD1* resulted in successful detection of mutations located in the duplicated regions (c.6727 C > T and c.5014_5015delAG, both located in exon 15), which could not be detected efficiently using WES. This coincides with the good coverage and RD produced using target enrichment over all coding regions of *PKD1*, including the problematic exons 1–32 and *PKD2*. One of the key differences between WES and target enrichment is gDNA library preparation and enrichment. For WES, sequence enrichment is achieved through an oligonucleotide probe-based capture strategy, whereas a PCR-based method is used for target enrichment^40–43^. While the oligonucleotide probe-based capture strategy for larger DNA regions is preferred over PCR-based methods for time and cost reasons, its capture efficiency is between 70–80%, as exons with high GC or AT content have reduced hybridisation and amplification efficiency^19,44^. In addition, a common limitation for capture-based enrichment methods is the presence of pseudogenes, which results in complications in variant calling and data interpretation of the targeted regions^19^. However, PCR enrichment methods are more efficient and reliable when analysing genes with pseudo-regions such as *PKD1*, as they have shown high specificity, sensitivity and reproducibility^45^. In addition, the ability to modify the design of probes in the PCR method provides another advantage, as this allows specific targeting of the functional gene rather than the pseudogenes, avoiding diagnostic limitations, including false negative results caused by low coverage of *PKD1* duplicated regions (as seen in WES) and false-positive results caused by the inability to avoid pseudogenes and mutations in these regions. Target enrichment of *PKD1* appears to overcome the limitations of WES and perhaps is more suitable, now, for ADPKD diagnostic applications.

In conclusion, the ability to effectively implement WES in current medical practices will improve care of patients by enhancing disease diagnosis and treatment planning. WES provides a rapid diagnostic tool for many genetic diseases and disorders, as it allows identification of common and novel genetic variants that may then be evaluated for their pathological impact. As for ADPKD, although the WES platform successfully identified novel *PKD1* mutations, it showed low sensitivity and RD of the duplicated regions, which represent a challenge for effective and reliable genetic diagnosis of ADPKD. WES enrichment strategies must be improved to solve the low sensitivity problem in the *PKD1* duplicated regions. Such enhancements would allow more rapid and accurate genetic analysis of ADPKD patients, which in turn would contribute to better disease management and improve our understanding of the molecular pathology underlying the disease.

## Methods

### Patient inclusion criteria

Six families with a history of ADPKD were selected from the Nephrology unit database at Mubarak Al-Kabeer Hospital, Kuwait for inclusion in the current study. ADPKD patients showed typical clinical presentations of ADPKD, including multiple renal cysts and impaired kidney function. Total 51 individuals from 6 families with typical ADPKD, including 26 ADPKD patients and 25 at-risk individuals were enrolled in the current study, which was reviewed and approved by the Joint Committee for The Protection of Human Subjects in Research of the Health Sciences Center (HSC) at Kuwait University and the Kuwait Institute for Medical Specialization (KIMS) (Reference: VDR/JC/690). Written informed consent was obtained from all patients before their enrollment in the study. All methods were performed in accordance with the guidelines of the joint HSC and KIMS ethical committee.

### Clinical evaluation

Clinical evaluation of all 51 subjects from the 6 families was performed. Individuals were evaluated using ultrasonographic analysis and renal function tests to confirm their disease status, including those showing negative results in mutation screening. Healthy individuals were later used as negative controls for comparative analysis with ADPKD patients.

### Abdominal ultrasound

All subjects enrolled in the current study except those who had undergone kidney transplants were instructed to fast for 6 h prior to abdominal ultrasound examinations, which were performed using a Logic 7 GE ultrasound with a curvilinear 3.5 MHZ probe. Focused ultrasound was performed to assess the kidneys, liver, pancreas and ovaries in female subjects. Each kidney was assessed in multiple views. Renal cysts in each kidney were examined and counted for diagnostic purposes according to the unified criteria for ultrasonographic diagnosis of ADPKD^22^. Each total kidney volume (TKV) was calculated using the ellipsoid formula: volume = length × lateral diameter × anterior-posterior diameter × π/6. TKV was calculated automatically by the machine in cubic centimetres (cc) and adjusted for height (htTKV expressed as cc/m)^46^. Each patient’s liver and pancreas were also screened for the presence or absence of cysts.

### Renal function test

From each subject, 5-mL blood samples were taken and used to conduct renal function test (RFT), which were performed at the Mubarak Al-Kabeer Hospital, Jabriya, Kuwait. Serum creatinine levels were determined for each patient and expressed as µ mol/L. Estimated glomerular filtration rates (eGFR) were calculated using the CKD-EPI creatinine equation (2009) developed by Levey *et al*.^47^. Calculations were performed using the GFR calculator provided on The National Kidney Foundation website www.kidney.org.

The RFT was not performed for ADPKD patients who reached ESRD, were on dialysis or had undergone a kidney transplant. For these patients, only abdominal ultrasound and genetic testing were performed to confirm ADPKD diagnosis, and their eGFR and htTKV were not included in the analysis.

### DNA isolation

From each subject, 10-mL blood samples were collected at the Nephrology Department of the Mubarak Al-Kabeer Hospital, Jabriya, Kuwait and genomic DNA was isolated from blood samples using a Gentra Puregene Blood Kit (Qiagen, 158467) following the manufacturer’s protocol.

### Mutation screening and classification of variants

#### Long-Range PCR

Mutations were screened in the genomic DNA of all subjects using locus-specific amplification and direct sequencing of exonic and flanking intronic regions of *PKD1* and *PKD2*^11^. Segregation was tested in family members using sequence analysis of the relevant genomic fragments. The significance of missense variants was assessed using the ADPKD Mutation Database (http://pkdb.mayo.edu), multi-sequence alignments and substitution assessment tools (SIFT, PolyPhen2 and Align GVGD), as previously described^11,20^ Novel frame-shifting insertions and deletions were defined as pathogenic.

#### Whole exome sequencing

DNA samples from 26 ADPKD patients from the 6 families were prepared and enriched using TruSeq v3, SureSlect v4 or v6 following the manufacturer’s protocol. Agilent’s QPCR NGS Library Quantification Kit (G4880A) was used to determine the DNA concentration of each library prepared. Enriched samples were pooled at a final concentration of 10 nM. Exome sequencing was performed using the Illumina HiSeq2000 platform. For mapping and alignment, read files (Fastq) were generated and obtained from the HiSeq2000 platform using the manufacturer’s proprietary software. Burrows-Wheeler Aligner package version 0.6.1^48^ was used to locate reads in the most recent map of the human genome (hg38/GRCh38). To ensure a minimum number of mismatching bases across the reads obtained, which in turn reduces false-positive SNP calls (indels), we utilised Genome Analysis Tool Kit (GATK) version 1.6^35^, which locally realigned mapped reads around potential insertion/deletion (indel) sites. Picard version 1.62 was used to label duplicate reads so as to remove those likely resulting from PCR bias. Generated BAM files were further manipulated using Samtools version 0.1.18^49^. To improve the quality of variants calls, GATK’s covariance recalibration was used to recalibrate base quality (Phred scale) scores. GATK Unified Genotyper was used to call SNP and indel variants in each sample^50^. Variant novelty was determined using dbSNP.

For variant calling and analysis, we used Golden Helix SNP & Variation Suite version 8.3.4 for Win64. We performed multiple sample variant calling to reduce calling sequencing errors and to enhance the accuracy and sensitivity of calling^51–53^. Filtered and unfiltered VCF files of all samples were uploaded and *Homo sapiens* (Human), GRCh37 g 1k (Feb2009) was used as the default genome assembly. *PKD1* (NM_001009944.2) and *PKD2* (NM_000297.3) variant annotations and analyses were performed using the dbNSFP Functional Predictions and Scores 2.9, GHI database^54–56^, which provided pathological prediction scores from prediction algorithms, including SIFT, Polyphen2, LRT, FATHMM and MetaLR; and conservation scores from phyloP100way_vertebrate, phastCons100way vertebrate, GERP++ and SiPhy and other function annotations, which were used to assess pathological impact. All non-synonymous and insertion/deletion variants were tested and validated in healthy and affected members of the ADPKD families using Sanger sequencing.

No filters were applied when calling variants to assess the quality of called variants in *PKD1* and *PKD2*. The quality was assessed by analysing the reading depth (RD), mapping quality (MQ), genotype quality (GQ), phred scale quality and quality by depth for each called variant. When the same variant was called in more than one sample, the mean of the quality measure was obtained and the standard deviation (STD) was calculated.

The *PKD1* and *PKD2* regions captured using WES were obtained by plotting BAM files against RefSeq genes 105v2, NCBI to highlight the gene regions. In addition, the total reads per exon in both genes were calculated for each sample and presented graphically.

#### Whole genome sequencing and analysis

DNA was extracted from blood samples using Qiagen Kits according to the manufacturer’s protocol. DNA quality and concentration were determined, and then 1 ug of the DNA was used for WGS using the Illumina TruSeq DNA sample preparation guide to obtain a final library of 300–400 bp average insert size. Covaris systems, which produces dsDNA fragments with 3′ and 5′ overhangs, was used to fragment genomic DNA that was then converted to have blunt ends using an end repair mix; the 3′ overhangs were removed using 3′ to 5′ exonuclease, and the 5′ overhangs were filled by the polymerase. The library size was selected using different ratios of the sample purification beads. Ligation was prevented by adding a single adenine nucleotide to the 3′ end of the blunted fragments, and the corresponding thymine nucleotide provided a complementary overhang on the 3′ end of the adapter. Multiple indexing adapters were ligated to both ends of the fragments in preparation for hybridisation in the Illumina flow cell (Illumina Hiseq2500). Isaac aligner software was used to align NGS sequencing data^57^. Resulting VCF files were analysed using golden helix software for the WES data.

#### Targeted enrichment arrays

Four samples with mutations in exons 1–32 of *PKD1* that were detected by LR-PCR but not WES were selected for this analysis. HaloPlex Custom kits (Agilent Genomics) were used to design panels covering *PKD1* and *PKD2* coding exons, such as 5′ UTR and 3′ UTR. The design was made for the Illumina platform using RefSeq, Ensembl, CCDS, Gencode, VEGA and SNP databases, and the designed panels were used according to the manufacturer’s protocol (G9900–90020). Generated BAM and VCF files were analysed using Golden Helix SNP & Variation Suite version 8.3.4 for Win64. *Homo sapiens* (Human), GRCh37 g 1k (Feb2009) was used as the default genome assembly. We eliminated all filters for the analysis to evaluate the quality of all called variants, which was assessed by analysing the RD, MQ, GQ, phred scale quality and quality by depth for each called variant.

#### Statistical analysis

To test the hypothesis that the slope for predicting htTKV levels from subject age is greater for ADPKD patients than for healthy subjects, linear regression analyses were performed on htTKV-age data from ADPKD patients and healthy subjects. This was followed by a comparison of the resulting regression slopes using Student’s *t*-test to evaluate the difference between the slopes. Similar analyses were also performed on the eGFR-age data.

#### Kaplan–Meier renal survival analysis

Survival times were calculated as the time of onset of ESRD, and a Kaplan–Meier product-limit survival curve was constructed using MATLAB and Statistics Toolbox, Release 2012b (The MathWorks, Inc., Natick, MA, USA). Twenty-three patients had already reached ESRD by the time of the analysis (some of the ESRD patients were part of the 6 families enrolled in the current study, but were excluded from the genetic testing as they died prior to screening). The median survival time was calculated as the smallest survival time for which the estimated probability of renal survival was ≤0.5. The mean survival time was calculated as the area under the Kaplan–Meier survival curve^58^.

## Supplementary information

Dataset 1
