# Supplementary material for: PKD1 Duplicated regions limit clinical Utility of Whole Exome Sequencing for Genetic Diagnosis of Autosomal Dominant Polycystic Kidney Disease
Source: Sci Rep. 2019 Mar 11;9:4141. doi: 10.1038/s41598-019-40761-w (PMC6412018; doi:10.1038/s41598-019-40761-w)
Supplement: Supplementary file 1 — Dataset 1 [file 41598_2019_40761_MOESM1_ESM.docx]

***PKD1* Duplicated regions limit clinical Utility of Whole Exome Sequencing for Genetic Diagnosis of Autosomal Dominant Polycystic Kidney Disease**

Hamad Ali^1,2,3^*, Fahd Al-Mulla^2*^, Naser Hussain^3^, Medhat Naim^3^, Akram Asbeutah^4^, Ali AlSahow^5^, Mohamed Abu-Farha^6^, Jehad Abubaker^6^, Ashraf Al Madhoun^2^, Sajjad Ahmad^7,8^ and Peter C. Harris^9^

(1) Department of Medical Laboratory Sciences, Faculty of Allied Health Sciences, Health Sciences Center, Kuwait University, Kuwait

(2) Department of Genetics and Bioinformatics, Dasman Diabetes Institute (DDI), Dasman, Kuwait

(3) Division of Nephrology, Mubarak Al-Kabeer Hospital, Ministry of Health, Kuwait

(4) Department of Radiological Sciences, Faculty of Allied Health Sciences, Health Sciences Center, Kuwait University, Kuwait

(5) Division of Nephrology, Al-Jahra Hospital, Ministry of Health, Kuwait

(6) ) Department of Biochemistry and Molecular Biology, Dasman Diabetes Institute (DDI), Dasman, Kuwait

(7) Department of Eye and Vision Science, Institute of Ageing and Chronic Disease, Faculty of Health and Life Sciences, University of Liverpool, Liverpool, United Kingdom

(8) St. Paul's Eye Unit, Royal Liverpool University Hospital, Liverpool, United Kingdom

(9) Division of Nephrology and Hypertension, Mayo Clinic, Rochester, USA

*Corresponding authors: [hamad.ali@hsc.edu.kw](mailto:hamad.ali@hsc.edu.kw) and fahd@al-mulla.org

**Supplementary Figures and tables**


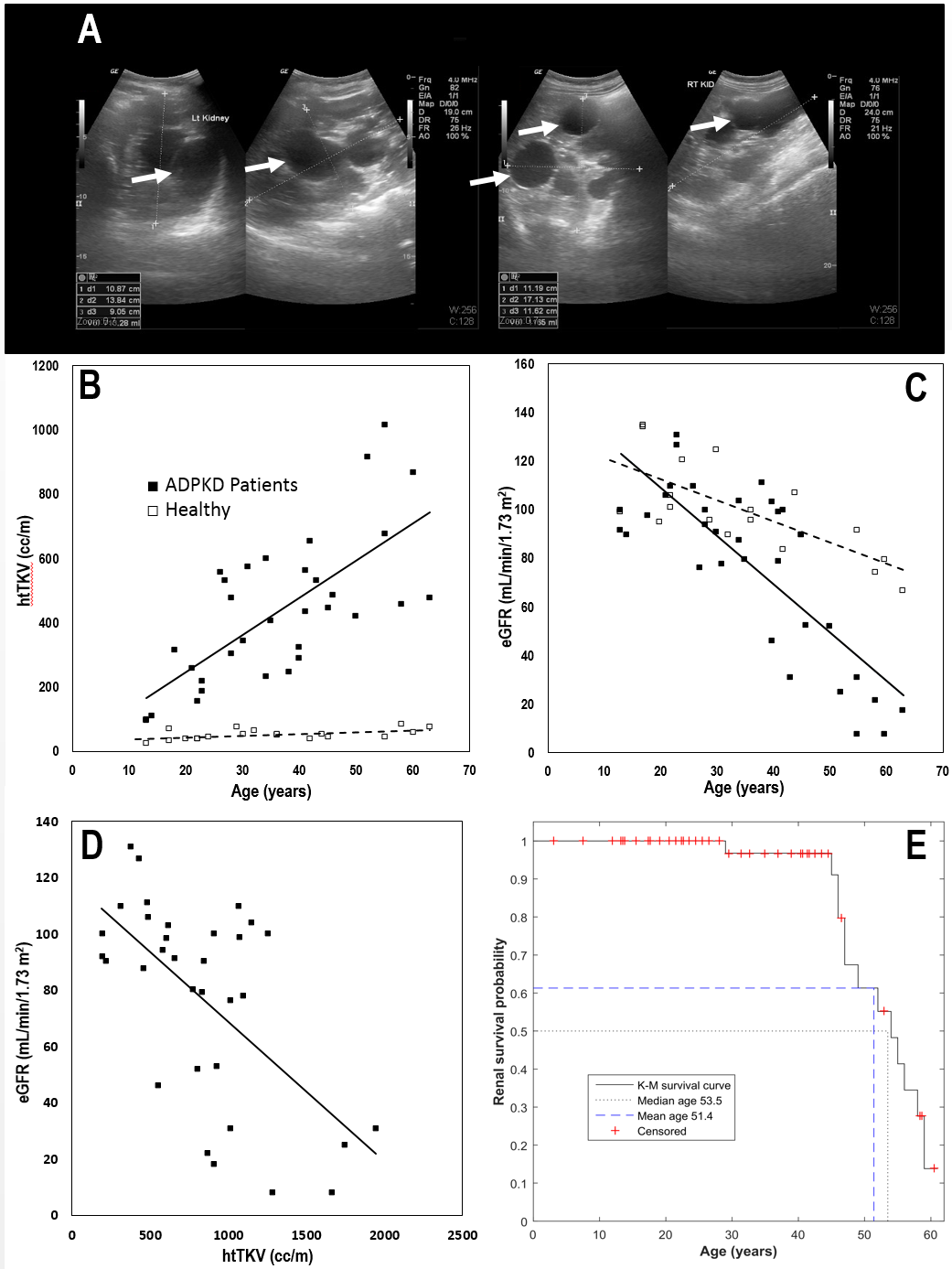


**Figure 1** Clinical evaluation of ADPKD patients. (A) Renal ultrasound analysis of ADPKD patient showing bilateral renal cysts which are indicated by the white arrows. (B-D) Correlation between height-adjusted total kidney volume (htTKV) and eGFR with age. Linear regression analyses indicated that ADPKD patients have a significantly higher rate of htTKV progression than healthy individuals (p<0.00001; compare slopes in Figure.1B). Patients with ADPKD also showed more progressive decrease of eGFR with age when compared to healthy individuals (p<0.002; compare slopes in Figure.1C). Calculated Pearson’s correlation coefficient indicated inverse relationship between htTKV and eGFR in ADPKD patients (-0.612) (Figure.1D). Out of 33 ADPKD patients, 13 have already reached ESRD by the time of consent. Kaplan-Meier renal survival analysis estimated the median of ESRD onset as 53.5 years and the mean age of ESRD as 54.4 years (Figure.1E).

Supplementary Table 1. Pathologic significance of PKD1 and PKD2 non-synonymous missense variants detected by WES

| WES Variant | Protein Change | SIFT Score  and Prediction^1^ | Polyphen2 HVAR Score  and Prediction^2^ | FATHMM Score and Prediction^3^ | MetaLR Score and Prediction^4^ |
| --- | --- | --- | --- | --- | --- |
| *PKD1* (NM_001009944.2) | | | | | |
| 16:2140294-SNV | p.Val4146Ile | 0.170  Tolerated | 0.879  Possibly damaging | 1.310  Tolerated | 0.144  Tolerated |
| 16:2140554-SNV | p.Ala4059Val | 0.44  Tolerated | 0.006  Benign | -0.340  Tolerated | 0.010  Tolerated |
| 16:2140680-SNV | p.Ile4045Val | 0.5  Tolerated | 0.003  Benign | -0.370  Tolerated | 0  Tolerated |
| 16:2141028-SNV | p.Ala3954Pro | 0.19  Tolerated | 0.943  Probably damaging | -0.550  Tolerated | 0.292  Tolerated |
| **16:2141795-SNV** | **p.Trp3842Arg** | **0**  **Damaging** | **0.999**  **Probably damaging** | **-3.370**  **Damaging** | **0.879**  **Damaging** |
| 16:2144176-SNV | p.Ala3512Val | 0.66  Tolerated | 0.001  Benign | 1.420  Tolerated | 0  Tolerated |
| 16:2144182-SNV | p.Thr3510Met | 0.049  Damaging | 0.107  Benign | 1.190  Tolerated | 0.005  Tolerated |
| 16:2152387-SNV | p.Phe3066Leu | 0.62  Tolerated | 0.466  Possibly damaging | 1.230  Tolerated | 0.000  Tolerated |
| 16:2158570-SNV | p.Arg2200Cys | 0.06  Tolerated | 0.854  Possibly damaging | -0.600  Tolerated | 0.295  Tolerated |
| 16:2164808-SNV | p.Arg739Gln | 1  Tolerated | 0  Benign | 1.270  Tolerated | 0  Tolerated |
| 16:2165470-SNV | p.Cys669Phe | 0.15  Tolerated | 0.178  Benign | 1.280  Tolerated | 0.051  Tolerated |
| *PKD2* (NM_000297.3) | | | | | |
| 4:88928968-SNV | p.Arg28Pro | 0.01  Damaging | 0.011  Benign | -0.160  Tolerated | 0  Tolerated |
| 4:88929453-SNV | p.Ala190Thr | 0.42  Tolerated | 0.444  Benign | -0.170  Tolerated | 0  Tolerated |
| 4:88967919-SNV | p.Phe482Cys | 0.02  Damaging | 0.586  Possibly damaging | -0.470  Tolerated | 0.340  Tolerated |
| 4:88989089-SNV | p.Met800Leu | 0.71  Tolerated | 0  Benign | -0.410  Tolerated | 0.113  Tolerated |

1. SIFT scores range from 0 to 1. Scores <0.05 are predicted to be damaging [1].
2. Polyphen2 HVAR score predictions as follows; Probably damaging (0.909-1), Possibility damaging (0.447-0.908) and Benign (0-0.446) [2].
3. FATHMN scores less than -1.5 are predicted as Damaging while scores higher than -1.5 are predicted as Tolerated.
4. MetaLR scores ranges from (0-1). Deleterious threshold >0.5 [3].

Highlighted variant is ADPKD mutation.

Supplementary Table 2. Conservation analysis of PKD1 and PKD2 non-synonymous missense variants detected by WES

| WES Variant | Protein Change | PhyloP 100way Vertebrate^1^ | GERP++ RS ^2^ | PhastCons 100way Vertebrate^3^ | SiPhy 29way ^4^ |
| --- | --- | --- | --- | --- | --- |
| *PKD1* (NM_001009944.2) | | | | | |
| 16:2140294-SNV | p.Val4146Ile | 2.055 | 4.020 | 0.369 | 16.309 |
| 16:2140554-SNV | p.Ala4059Val | 0.472 | 1.250 | 0.127 | 4.533 |
| 16:2140680-SNV | p.Ile4045Val | -0.085 | -1.550 | 0.127 | 0.963 |
| 16:2141028-SNV | p.Ala3954Pro | 3.272 | 2.040 | 0.714 | 3.770 |
| **16:2141795-SNV** | **p.Trp3842Arg** | **7.201** | **4.490** | **0.714** | **13.940** |
| 16:2144176-SNV | p.Ala3512Val | 0.663 | -9.010 | 0.059 | 9.751 |
| 16:2144182-SNV | p.Thr3510Met | 1.263 | 0.474 | 0.127 | 9.165 |
| 16:2152387-SNV | p.Phe3066Leu | 0.754 | -2.710 | 0.313 | 4.659 |
| 16:2158570-SNV | p.Arg2200Cys | 1.064 | 3.340 | 0.127 | 10.309 |
| 16:2164808-SNV | p.Arg739Gln | 0.091 | -2.550 | 0.059 | 3.057 |
| 16:2165470-SNV | p.Cys669Phe | 2.692 | 3.190 | 0.272 | 4.542 |
| *PKD2* (NM_000297.3) | | | | | |
| 4:88928968-SNV | p.Arg28Pro | 0.516 | 0.550 | 0.261 | 2.045 |
| 4:88929453-SNV | p.Ala190Thr | 3.010 | 4.070 | 0.714 | 15.017 |
| 4:88967919-SNV | p.Phe482Cys | 6.099 | 5.610 | 0.388 | 15.780 |
| 4:88989089-SNV | p.Met800Leu | 1.491 | 3.070 | 0.339 | 12.700 |

1 Phylop 100way Vertebrate: Negative scores indicate faster-than expected evolution, while positive values indicate conservation. Deleterious threshold > 1.6

2 GERP++ RS: Deleterious threshold > 4.4

3 PhastCons 100way Vertebrate: scores range from 0-1. Scores higher than 0.5 are more likely to be conserved [4].

4 SiPhy 29way: Deleterious threshold > 12.17

Highlighted variant is ADPKD mutation

**References**

1. Ng PC, Henikoff S: **SIFT: Predicting amino acid changes that affect protein function**. *Nucleic acids research* 2003, **31**(13):3812-3814.

2. Adzhubei IA, Schmidt S, Peshkin L, Ramensky VE, Gerasimova A, Bork P, Kondrashov AS, Sunyaev SR: **A method and server for predicting damaging missense mutations**. *Nature methods* 2010, **7**(4):248-249.

3. Dong C, Wei P, Jian X, Gibbs R, Boerwinkle E, Wang K, Liu X: **Comparison and integration of deleteriousness prediction methods for nonsynonymous SNVs in whole exome sequencing studies**. *Human molecular genetics* 2015, **24**(8):2125-2137.

4. Prakash A, Tompa M: **Measuring the accuracy of genome-size multiple alignments**. *Genome biology* 2007, **8**(6):R124.
